# Supplementary material for: Equilibrium phase diagrams of isostructural and heterostructural two-dimensional alloys from first principles
Source: iScience. 2022 Mar 25;25(4):104161. doi: 10.1016/j.isci.2022.104161 (PMC9010766; doi:10.1016/j.isci.2022.104161)
Supplement: Document S1. Figures S1 and S2 [file mmc1.pdf]

iScience, Volume *25*

Supplemental information

Equilibrium phase diagrams of isostructural and heterostructural two-dimensional alloys from first principles

John Cavin and Rohan Mishra

Supplemental Information

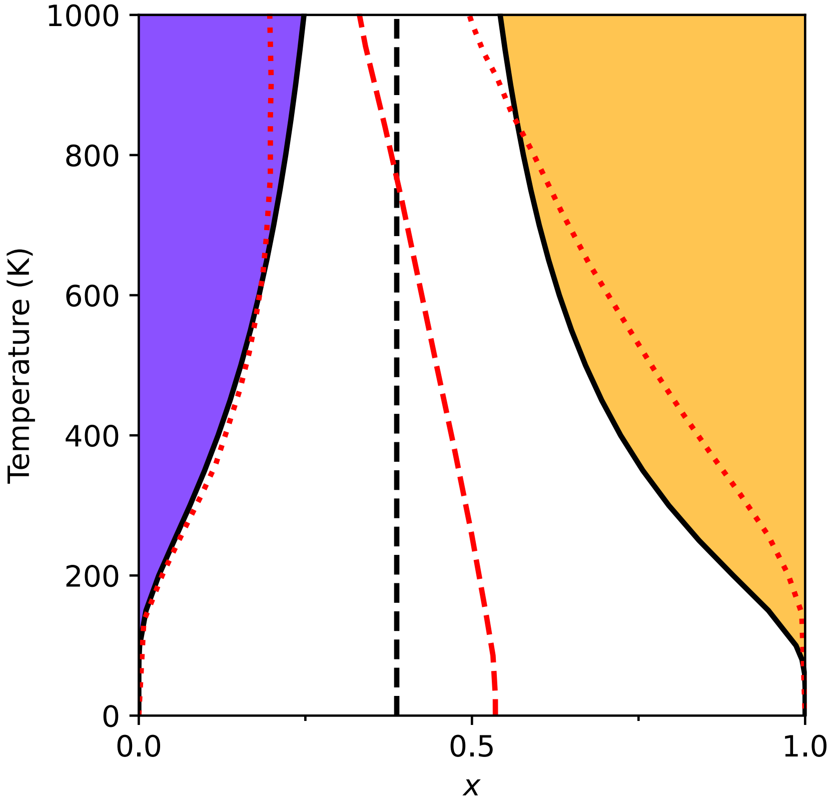

Figure S1. Phase diagram of $Mo_{1-x}W_xTe_2$ overlayed in red with non-spin orbit coupling, fixed-lattice phase diagram from Duerloo et al. The dotted red lines correspond to the boundaries between the *1T′* and *2H* phases and the metastable region. The red dashed line corresponds to the crossover boundary in the metastable region between the two phases. Adapted with permission from Duerloo, K.-A.N., and Reed, E.J. (2016). © 2016 American Chemical Society. See Key Resources Table and Figure 4.

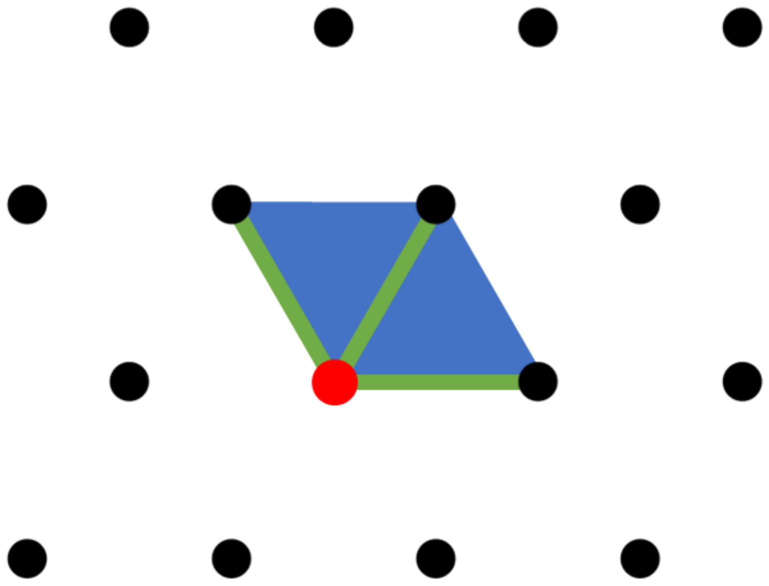

Figure S2. Diagram of a triangular lattice. The single red dot represents one one-body energy contribution per atom, the three green lines represent three two-body energy contributions per atom, and the two blue triangles represent two three-body energy contributions per atom. See METHOD DETAILS in STAR methods.
